# Isolation of Methanotrophic Consortium from Chernevaya Taiga Soil and Laboratory Research on Its Introduction into Agro-Soil

**DOI:** 10.3390/microorganisms13092052

**Published:** 2025-09-03

**Authors:** Irina K. Kravchenko, Liana G. Gogmachadze, Aleksei O. Zverev, Marina V. Sukhacheva, Alla L. Lapidus

**Affiliations:** 1 Winogradsky Institute of Microbiology, Research Center of Biotechnology, Russian Academy of Sciences, 119071 Moscow, Russia; lya.gogmachadze@yandex.ru; 2 Faculty of Soil Science, Moscow State University, 119192 Moscow, Russia; 3 All-Russian Research Institute for Agricultural Microbiology (ARRIAM), 196608 Saint Petersburg, Russia; 4 Skryabin Institute of Bioengineering, Research Center of Biotechnology, Russian Academy of Sciences, 119071 Moscow, Russia; msukhacheva@mail.ru; 5 Independent Researcher, 125493 Moscow, Russia

**Keywords:** methanotrophs, methane oxidation, Chernevaya taiga soil, microbial inoculation

## Abstract

Aerobic soils serve as significant sinks for atmospheric methane, with their effectiveness influenced by the diversity and activity of soil methanotrophs. Land-use changes, particularly the conversion of natural ecosystems to agriculture, can substantially alter these microbial communities. A promising strategy to restore methane oxidation capacity is the introduction of active, ambient methane-oxidizing bacteria. The stable methane-oxidizing microbial consortium T1, dominated by *Methylocystis* (74%), was isolated from the soil of the unique Chernevaya Taiga forest ecosystem. The effects of inoculating this consortium were evaluated in a four week laboratory incubation experiment, using microcosms of soddy-podzolic agro-soil. Methane oxidation potential was assessed to measure methanotroph activity; methanotrophs were quantified using qPCR targeting *pmo*A genes; and the diversity of soil microbial communities was examined through 16S rRNA gene profiling. Inoculated soils exhibited significantly higher methane oxidation potentials compared to non-inoculated soils. Furthermore, *pmo*A gene copy numbers in the inoculated soils were significantly elevated (10^6^ copies *pmo*A g^−1^), indicating stable persisted methanotrophic populations throughout the incubation period. These findings suggest that enriched methanotrophic consortium inoculation into agro-soils may be a promising strategy for restoring methane-oxidizing activity.

## 1. Introduction

Methane (CH_4_) is recognized as an important greenhouse gas, having a global warming potential that is significantly higher than carbon dioxide (CO_2_) over shorter time horizons (e.g., 7–12 years). This means that even though methane’s atmospheric lifespan is shorter than CO_2_’s, its ability to trap heat is considerably greater, making it a critical target for climate change mitigation efforts [1]. Both natural (wetlands, thawing permafrost) and man-made (agricultural, fossil fuel production, waste management) sources emit methane into the atmosphere [2,3]. Consequently, mitigating methane emissions is crucial for achieving long-term climate objectives and diminishing the short-term rate of global warming.

Methane mitigation techniques must be developed and implemented, due to the rapid rise in the atmospheric concentration of this powerful greenhouse gas. While controlling methane emissions is crucial to lower the rate of global warming, surprisingly little is known about methane-decreasing programs and their effectiveness. Although the bulk of known methane mitigation strategies focus on reducing emissions, it has been estimated that only 13% of global methane emissions are addressed by direct methane reduction techniques [4].

Methanotrophs that use methane as a carbon and energy source contribute to the effective removal of methane when special biofilters are used at landfills, wastewater treatment plants, and coal mines [5]. Methane is naturally converted by these bacteria into CO_2_ and biomass, which are valuable byproducts. Using aerobic methanotrophic bacteria, which can eliminate methane at low concentrations, is one prospective methane mitigation strategy. Although some methanotrophs have been seen to develop oxidizing ambient methane, carbon monoxide, and hydrogen over a 12 month period, the current quantity of atmospheric methane (1.9 ppm) makes it impossible for aerobic methanotrophs to survive [6].

Forest soils, which are frequently disregarded in discussions about methane cycling, are an important methane sink, and this natural process plays a crucial role in regulating the amount of methane in the atmosphere [7]. A varied and active community of microorganisms, particularly methane-oxidizing bacteria (methanotrophs), is responsible for this capacity [8]. Because of particular climatic conditions, such as restricted nitrogen supply and suitable moisture content, which lead to considerable methane consumption, methanotrophs flourish in the upper layers of forest soils.

The natural balance that supports methane oxidation in forest soils is increasingly threatened by the conversion of forest lands for agricultural purposes. Driven by the growing global demand for food, deforestation and the conversion of forest lands into agricultural fields are becoming widespread practices. This land-use change drastically alters the soil environment, disrupting the established microbial communities and impacting the methane oxidation process [9]. Agricultural practices, such as tilling, fertilization, and irrigation, drastically change the soil structure, nutrient availability, and moisture content, creating conditions that are less favorable for methanotrophs and potentially favoring methane-producing bacteria (methanogens) [10]. As forest lands are converted to agricultural areas, the total area capable of acting as a methane sink diminishes, leading to a net increase in methane emissions [11,12]. The loss of forest soil’s methane oxidation capacity and the simultaneous increase in methane emissions from agricultural activities create a dangerous feedback loop, accelerating the rate of global warming [13].

Chernevaya Taiga is a unique forest ecosystem located in Western Siberia, characterized by extreme humidity, special soil types, and unusual plant species [14,15]. According to Abakumov et al. [14] and Kravchenko et al. [16], it is well-known for its high microbial activity in relation to the cycles of crucial biogenic components like carbon and nitrogen. This suggests a complex and dynamic microbial community, adapting to specific environmental constraints. Despite the ecological significance of the Chernevaya Taiga and its potential role in methane cycling, studies specifically focusing on methane oxidation and the identification/characterization of methanotrophic communities within this ecosystem are not currently available. This lack of research represents a significant knowledge gap, hindering our understanding of the overall contribution of Siberian forests to methane mitigation.

Novel methane-oxidizing microbes and their microbial consortia, sourced from the unique soils of the Chernevaya taiga ecosystem, offer promising potential as bioinoculants in cutting-edge ecobiotechnological applications. In methane-rich environments like rice paddies and landfills, the introduction of methanotrophic bacteria has proven effective in mitigating methane emissions. In addition to improving paddy production and growth indices, field studies demonstrated that the isolated methanotrophic strain MR1 effectively reduced methane emissions [17,18]. Inoculating biogas digestion effluent with *Methylosinus* and *Methylocystis* markedly reduced methane emissions from rice paddies when the effluent was applied as an organic fertilizer [19]. Similarly, the application of *Methylocystis* sp. JHTF4 and *Methyloversatilis* sp. JHM8 during rhizoremediation of diesel-contaminated soils demonstrated significant efficacy in lowering CH_4_ emissions [20]. The application of combined methanotrophic enrichments to landfill cover soils led to a measurable reduction in methane emissions from the landfill surface [21].

The aim of this study is to investigate methanotrophic bacteria in the Chernevay taiga soil to assess their possible use to increase methane oxidation activity when applied to agricultural soils. This study represents the first investigation into methanotrophic bacterial communities within the soils of the Chernevaya taiga ecosystem. The primary objective of this investigation is to explore the following key questions: (i) Can the Chernevaya taiga soil contribute to the oxidation of atmospheric methane? (ii) Is it possible to isolate an active, highly enriched methanotrophic consortium from this soil? (iii) Will the isolated consortium be able to maintain its viability and methane oxidation activity when incorporated into an agro-soil in another geographical area?

We hypothesize that the methanotrophs found in Chernevaya taiga soil have unique environmental adaptations and can be employed as bioinoculants for the restoration processes that convert methane to carbon dioxide and reduce its emissions in agricultural soil. The outcomes could serve as the basis for developing an ecological in situ biotechnology that mimics nature and controls methane emissions from anthropogenically degraded ecosystems.

In order to test our hypotheses, we incubated oxic agro-soil microcosms for 28 days, inoculating them with the methanotrophic consortium and isolating it from Chernevaya taiga soil. To measure the efficiency of inoculation we monitored the potential rate of methane oxidation; to assess the stability of introduced consortium, we evaluated the bacterial genes number (16S and *pmo*A) and 16S rRNA amplicon sequencing of soil microbial communities.

## 2. Materials and Methods

### 2.1. Sample Source, Determination of Soil Methanotrophic and Respiration Activity

In our studies we have used samples of dark gray soil (Retisols, FAO classification) of Chernevaya taiga (56.30693° N, 85.47063° E) under a tallgrass fir–aspen forest with a shrinking fir stand. The region experiences a moderately continental climate, characterized by prolonged winters and pronounced fluctuations in both daily and annual temperatures. The mean average annual air temperature (MAAT) is 1.75 °C, average temperature in July is 17 °C, and the average temperature in January is −19–21 °C. Despite the prolonged winter, the soil remains unfrozen throughout the season, insulated by a substantial snow cover. The frost-free period is 100–105 days.

Field studies were conducted in May 2020 in the Tomsk region, Russia. Soil samples were stored in an air-dry state until the start of the experiments in June 2024. We aimed to obtain drought-resistant methanotrophic consortia that successfully integrate into the native microbial community of agricultural soil with a seasonal drying–rewetting cycle. Prior publications provide a thorough description of the regions, study locations, and soil parameters [14,15].

A gas chromatography (GC) headspace method was applied for soil potential CH_4_ oxidation activity and soil microbial respiration (CO_2_ emission) analysis after a 24 h incubation of a rewetted soil according to a previously established procedure [22]. Briefly, 5 g of soil was placed in a 20 mL glass vial and rewetted with 1.25 mL of deionized water, before being sealed with a butyl septa and metal collar. For respiration activity the samples were incubated at room temperature (22 °C) for 24 h. The amount of CO_2_ released from the soil was determined by comparing the headspace in the ambient vial with the headspace above the soil following incubation.

Methane at a concentration of approximately 200 ppm was injected into the gas phase to evaluate its oxidation. We used a gas chromatograph (GC) to determine the actual methane concentrations. The vials were incubated at 25 °C for 24 h, gas samples (0.25 mL) were periodically sampled from the headspace, and methane concentrations were measured. The activity was calculated based on the linear regression of the methane concentration decreasing with incubation time, the volume of the GC vials, and the moisture content of the incubated soils and expressed in ng CH_4_ g.d.w.^−1^ h^−1^.

The concentration of CH_4_ and CO_2_ in the gas phase was measured using a Cristall 5000.1 gas chromatograph (Chromatek, Yoshkar-Ola, Russia) equipped with a flame ionization detector on a 3.0 m × 0.2 cm column filled with HayeSep N. The temperature of the column was 40 °C, and the detector was 200 °C. Argon, which served as a carrier gas, was supplied at a rate of 25 mL/min.

### 2.2. Isolation of Methane-Oxidizing Consortium from Chernevaya Taiga Soil

The methane-oxidizing consortium T1 was enriched and isolated using liquid mineral media «P» [23], which was diluted five times (P/5).

Soil samples were subjected to serial ten-fold dilutions and 0.1 mL was inoculated into 20 mL flasks containing 5 mL of P/5 medium. Methane was introduced into the gas phase of each flask by adding it with a syringe fitted with a filter tip, reaching a final concentration of 10% by volume. The vials were incubated for 14–30 days at 25 °C on a PSU-20i orbital shaker (BioSan, Riga, Latvia), operating at 150 rpm, until the optical density at 600 nm (OD_600_) reached 0.2–0.3. Subsequently, 0.1 mL of the suspension was transferred to a fresh vial and incubated under the same conditions as previously described. This serial transfer was repeated eight times until stable growth and consistent microscopic morphology were observed for nine enriched consortia. Live-cell imaging was conducted using phase contrast microscopy with a Leitz Optilux microscope (Leica Microsystems, Wetzlar, Germany).

### 2.3. Microcosm Experiments

Microcosm experiments were conducted to assess methane oxidation activity, monitor the dynamics of inoculated methanotrophs, and evaluate shifts in native soil microbial communities.

Soil samples were collected on 5 June 2024, from the agrogenic soils of herb-bunchgrass meadows near the Petelino poultry production facilities in the Odintsovo district, Moscow Region, Russian Federation (50°36′33″ N, 36°49′39″ E). According to Russian soil classification, the soil is categorized as soddy-podzolic, heavy loamy on moraine deposits. Under the FAO classification system, it is classified as Retisol, while the World Reference Base for Soil Resources (WRB) classifies it as Eutric Albic Retisol (Abruptic, Loamic).

To ensure successful randomization in our laboratory microcosm experiments, each microcosm was prepared identically and then randomly assigned to a specific treatment group (control or inoculation) and cultivation duration (0, 1, 2, or 4 weeks). This approach minimized experimental bias and accounted for potential spatial variability.

**Figure 1:**
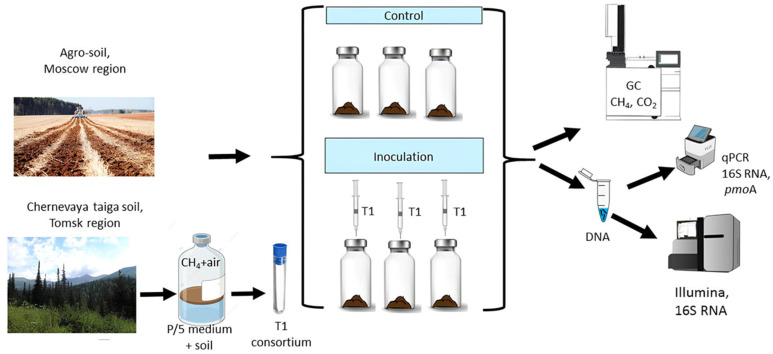
Diagram of experimental setup on agro-soil microcosm laboratory incubations.

The experiment utilized two treatments: (a) nine control microcosms containing native soil (C); and (b) eighteen microcosms inoculated with the microbial consortium (0.5 mL of a bacterial suspension containing 10 × 10^9^ cells/mL) (Figure 1). Inoculated microcosms were labeled according to incubation periods: 0, 1, 2, and 4 weeks. Each microcosm comprised 10 g of soil in a 120 mL glass bottle. Microcosms were incubated in the dark at 25 °C for four weeks, with soil moisture maintained at 50% water holding capacity (WHC) throughout the experimental period.

Three microcosms from each treatment were used to track methanotrophic activity and soil respiration dynamics at weeks 0, 1, 2, and 4. Concurrently, three other microcosms were sacrificed at the same time points for destructive DNA extraction. The characteristics of the indigenous soil microbial community were assessed in the control variant.

### 2.4. DNA Extraction, Quantitative PCR and Illumina 16S rRNA Sequencing

DNA was extracted from 2 mL of T1-consortium and 0.25 g of soil samples using a Power Soil Isolation Kit (Qiagen, Carlsbad, CA, USA), following the manufacturer’s recommendations. Each sub-sample from the same soil underwent independent extraction. The quantity and quality of the extracted DNA were assessed using a Nanodrop 1000 Spectrophotometer (Thermo Fisher Scientific, Waltham, MA, USA) and monitored on a 1% agarose gel.

Real-time quantitative PCR (qPCR) was performed in triplicate for each DNA sample to quantify total methanotrophic bacteria (MOB) and overall bacterial communities. Quantification was achieved using the A189f–A682r [24] and EUB338f–EUB518r primer sets, respectively. The PCR protocol consisted of 30 cycles with an annealing temperature of 56 °C. A pure culture of *Methylosinus trichosporium* OB3b served as the positive control, while a non-template control (NTC) was used as the negative control. Standard curves were established by performing serial dilutions of the positive control’s purified PCR product, ranging from 10^2^ to 10^8^ gene copies per microliter (μL^−1^). Assays were run on a CFX96 Real-Time PCR Detection System (Bio-Rad, Des Plaines, IL, USA). The reaction mixtures contained a ROX passive reference dye and SYBR GreenI (Syntol, Moscow, Russia). To verify the specificity of the amplicons, melting curves were generated at the conclusion of each run by increasing the temperature from 50 to 95 °C in 0.5 °C increments. Amplification specificity was confirmed by analyzing the melting curve for a single, distinct peak. All reactions were run in triplicate, and the mean and standard deviation (±SD) were reported for each sample.

For the taxonomic analysis of the bacterial community, we used universal primers F515/R806 to target the variable V4 region of the 16S rRNA gene. These primers (GTGCCAGCMGCCGCGGTAA/GGACTACVSGGGTATCTAAT) are specific to a wide range of microorganisms, including both bacteria and archaea [25].

A 15 µL reaction mixture was prepared for PCR, containing 0.5–1 unit of Q5^®^ High-Fidelity DNA Polymerase (NEB, Ipswich, MA, USA), 5 pM of each primer, 10 ng of DNA template, and 2 nM of each dNTP (Life-Technologies Corporation, Carlsbad, CA, USA).

The reaction was initially denatured at 94 °C for 1 min. This was followed by 35 cycles of: denaturation at 94 °C for 30 s; annealing at 50 °C for 30 s; and extension at 72 °C for 30 s. A final extension step was carried out at 72 °C for 3 min. PCR products were then purified using AMPureXP (Beckman Coulter, Indianapolis, IN, USA) as per the Illumina protocol.

Library preparation was performed in accordance with the manufacturer’s protocol, as detailed in the Illumina MiSeq Reagent Kit Preparation Guide (https://support.illumina.com/content/dam/illumina-support/documents/documentation/chemistry_documentation/16s/16s-metagenomic-library-prep-guide-15044223-b.pdf) (https://support.illumina.com/downloads/16s_metagenomic_sequencing_library_preparation.html, accessed on 20 May 2024) Sequencing was then carried out on an Illumina MiSeq device with a MiSeq^®^ Reagent Kit v3 (Illumina, Inc, San Diego, CA, USA) (600 cycle), employing a paired-end sequencing strategy (2 × 300 bp). All sequencing was conducted at the ARRIAM resource center for “Genomic Technologies, Proteomics, and Cell Biology.”

Sequences were quality-trimmed using the dada2 filtering approach [26]. The maximum number of expected errors was set to 2, and reads were truncated at 240 bp. Primers were removed prior to filtering and reads matching the phiX genome were discarded. The rarefaction threshold was set to the minimum sequencing depth per sample (17,591 reads).

### 2.5. Bioinformatics Analysis

Initial data processing, including demultiplexing and adapter removal, was performed using MiSeq System Suite (v.4.1.0) Illumina software. For all subsequent analysis, we used the R software environment. We used the dada2 [26], phyloseq [27], and DECIPHER [28] packages, along with the SILVA ribosomal DNA database release 138 [29], to denoise the data, merge sequences, remove chimeric reads, identify amplicon sequence variants (ASVs), and perform taxonomic classification. The results of the taxonomic analysis were visualized using the QIIME2 (v.2024.5)software package [30].

The general processing of sequences was carried out in the dada2 (v1.14.1) package [26], according to the author’s recommendations. Taxonomy annotation was performed using Naive Bayesian Classifier and SILVA 132 as the reference database [29]. Data analysis was conducted in R (v4.3.0) to assess microbial community structure and diversity metrics. The phyloseq (v1.30.0) [27] and tidyverse (2.0.0) [31] packages were used to create alpha- and beta-diversity plots, bar graphs, and heatmaps. We performed a PERMANOVA analysis with the vegan package (2.5–6) [32] to test for significant differences between groups. Differential abundance analysis was performed using DESeq2 to identify significantly enriched taxa across treatment comparisons [33]. Amplicon sequence variants (ASVs) were considered ‘variable’ if they met the following criteria: baseMean ≥ 50, log_2_ fold change ≥ 2, and adjusted *p*-value < 0.05.

The raw sequences are available on the SRA under accession number PRJNA1285408 (https://www.ncbi.nlm.nih.gov/sra/PRJNA1285408) (accessed on 2 July 2025).

### 2.6. Statistical Analysis

Laboratory experiments were conducted using three biological replicates, each representing independent samples derived from distinct microcosms. Although this is the minimum number recommended to provide a basic level of replication, it is sufficient in this study due to the completely randomized design, the expected magnitude of the treatment effects and the balancing with practical limitations, such as time, space, and cost of resources.

All assays were conducted in triplicate, and results are presented as the mean ± standard deviation (SD) from three independent experiments. Statistical analysis was carried out using Microsoft Excel and SPSS Statistics v. 17.0. A *p*-value of <0.05 was used as the threshold for statistical significance in all tests.

## 3. Results

### 3.1. Methane Oxidation and Methanotrophs Detection in Chernevaya Taiga Soil Samples

Over six days, methane added to the headspace of vials with soil was completely oxidized and the calculated potential methane oxidation rate in Chernevaya taiga soil samples was 124 ± 10 ng C-CH_4_ g d.w.^−1^ day^−1^.

In order to identify potential participants in the methane oxidation process, an analysis of the microbial community composition was conducted using 16S rRNA profiling. The average number of reads per sample was 149,446. The composition of the dominant phyla in the microbial communities of chernevaya soil was represented by Proteobacteria (26.96%), Actinobacteria (22.69%), and Verrucomicrobia (19.52%). Moreover, a significant portion was made up of representatives of Gemmatimonadota (9.70%), Chloroflexi (8.26%), Firmicutes (4.90%) and Bacteroidota (1.6%).

The number of species-level OTUs determined at 97% sequence identity was 580 and the most abundant OTUs are listed in Table 1. It should be noted that the most abundant OTUs were represented by the uncultivable bacterial taxa.

Only two OTUs of known methanotrophs were found in chernevaya soil samples. One phylotype of the methanotroph bacteria may be classified as a member of the genus *Methylocystis*. The 16S rRNA gene sequence showed 98.64% identity with 99% query cover to *Methylocystis echinoides* strain 2 (J458502.1), strains *Methylocystis* sp.DWT (AJ868423.1) isolated from soil; SB2 (GU734136.1) from spring bog; Qz-R (LC849105) from paddy soil. Another may be affiliated with *Methylocella* with 92% identity with type strain of *M. silvestris*. Considering the total microbial community, known methane-oxidizing bacteria constituted 0.04% of soil bacteria with 0.03% of *Methylocystis* and 0.01% of *Methylocella*. No OTUs belonging to *Beijerinckiaceae* bacteria, which could potentially be methanotrophs, were detected in chernevaya soil.

At the same time, quite numerous representatives of facultative methylotrophic bacteria were found. The most numerous components were *Methyloceanibacter* (3.1% of all bacterial 16SrRNA gene fragments) as well as a low relative abundant *Hyphomicrobium* (0.11%) and unc. *Methyloligellaceae* (0.01%).

The presence of a low abundance of methanotrophs in soils exhibiting high methane oxidation activity indicates that these bacterial populations may possess a high metabolic efficiency for methane consumption. This could be due to several factors, including the presence of specialized methanotrophs with high methane affinity, favorable environmental conditions, or a community structure that enhances methane oxidation. Some methanotrophs, particularly Type II methanotrophs, are known for their high affinity for methane, meaning they can effectively oxidize methane even at low concentrations. If the soil environment selectively supports highly active methanotrophs, even a small population may drive substantial methane oxidation.

### 3.2. Taxonomic Composition of T1 Methane Oxidizing Consortium Based on 16S rRNA Gene Analysis

**Figure 2:**
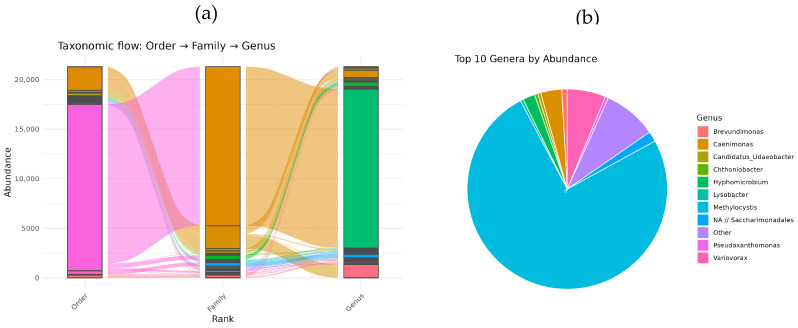
Composition of T1-consortium based on Illumina sequencing at different taxonomic ranks (**a**) and bacterial components at the genus level (**b**).

Illumina sequencing revealed that the T1 methane-oxidizing consortium, isolated from the soil of the Chernevaya Taiga, comprises a multi-component community-encompassing bacteria from various taxonomic groups (Figure 2a). At the order level, seven distinct orders were identified, with *Rhizobiales* being the most dominant. At the family level, nine families were detected, with *Beijerinckiaceae* being the most prevalent.

At the genus level, *Methylocystis* bacteria were the absolute dominants, constituting up to 74% of the total 16S rRNA gene fragments. Three *Methylocystis* ASVs were detected in consortium: dominant ASV31 and minor ASV1471 and ASV1745. Other notable genera included *Variovorax*, *Caenimonas*, *Hyphomicrobium*, *Lysobacter*, *Brevundimonas*, and *Chthonibacter* (Figure 2b).

### 3.3. Dynamics of Methane Oxidation and Soil Microbial Respiration Activity in Microcosm Experiments

The dynamics of methane oxidation and soil microbial respiration activity were evaluated under controlled conditions to understand how inoculation with the T1 methane-oxidizing consortium influences these microbial processes.

**Figure 3:**
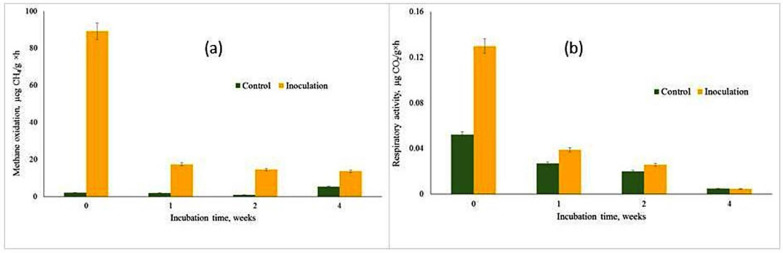
Dynamics of potential methane-oxidizing (**a**) and respiration activity (**b**) in soil microcosms. Data are presented as means with standard error bars (n = 3), indicating the variability and precision of the measurements.

A significant effect of inoculation with the T1 methane-oxidizing consortium on potential CH_4_ oxidation was observed at all sampling dates (Figure 3a). Immediately after inoculation, methane oxidation activity in the inoculated soil (0—week variant) was measured at 89.2 ng CH_4_· g dry weight^−1^ h^−1^, compared to 2.1 ng in the control soil.

Over the course of the incubation, methanotrophic activity in the inoculated samples declined to levels ranging from 17.4 to 13.7 ng CH_4_ g dry weight^−1^ h^−1^. Despite this decrease, the activity remained significantly higher than that observed in the control soil during the same period, which ranged from 0.9 to 5.2 ng CH_4_ g dry weight^−1^ h^−1^.

Inoculating soil with the methane-oxidizing consortium T1 initially enhanced methane oxidation rates, but with time this activity decreased. Changes in the composition of the microbial population, substrate depletion, and the impact of external factors may be responsible for this reduction. Long-term sustainability of the initial enrichment of particular mehanotrophs may be threatened by shifts in the dominant methanotrophs or the development of additional microorganisms, which could alter the structure of the microbial community.

Soil CO_2_ production, used as a proxy for microbial activity, varied across the four sampling dates (Figure 3b). Between-date variations in CH_4_ oxidation were positively correlated with CO_2_ accumulation (R^2^ = 0.68), indicating a strong relationship between these two processes. Since CO_2_ is a byproduct of methane oxidation, this correlation further supports the high activity of the introduced T1 methane-oxidizing consortium in enhancing methane oxidation rates.

### 3.4. The Abundance of Bacterial Genes in the Soil Samples from the Incubation Experiment, Determined Using Quantitative Polymerase Chain Reaction (qPCR)

**Figure 4:**
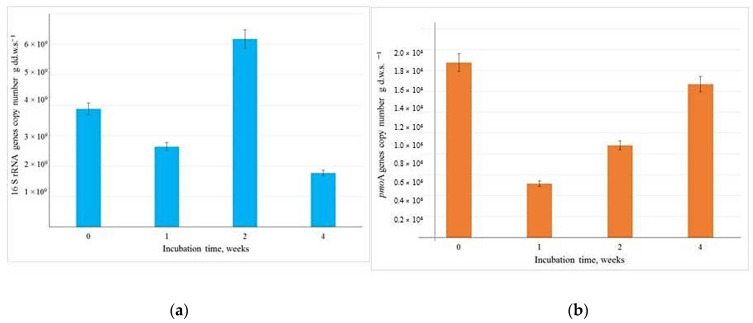
Abundance of 16 S rRNA (**a**) and *pmo*A (**b**) bacterial genes in soil microcosms inoculated with T1 mehanotrophic consortium. Data are presented as means with standard error bars (n = 3).

Our findings reveal that the number of 16S rRNA gene copies in the inoculated soil reached 3.9 × 10^9^ per gram of dry weight and exhibited minimal variation over the four week observation period (Figure 4a).

In contrast, the number of *pmo*A gene copies remained consistently high, fluctuating between 8.81 × 10^5^ and 1.47 × 10^6^ per gram of dry weight (Figure 4b). This indicates a significant and stable presence of methanotrophs within the soil microbial community, following the introduction of the T1 methane-oxidizing consortium. In the original control soil, the abundance of *pmo*A gene copies did not exceed 10^3^ per gram of soil.

### 3.5. Investigation of the Effect of Inoculating T1 Consortium on Soil Bacterial Community Diversity and Composition

**Figure 5:**
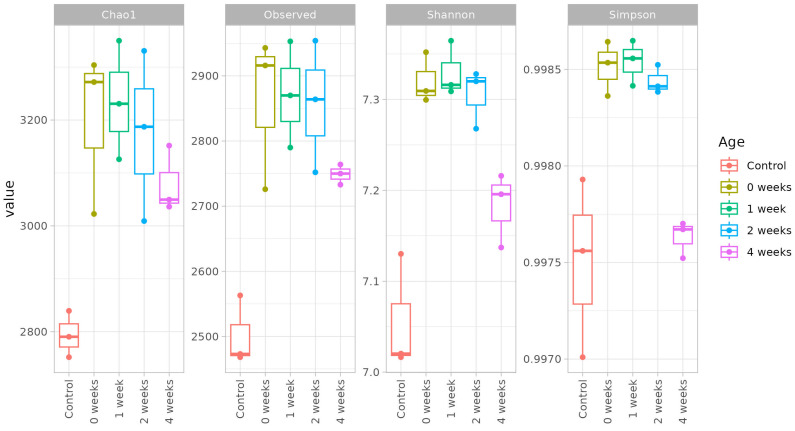
Indices of bacterial richness (Chao1. Observed), evenness (Shannon), and diversity (Simpson) in soil microcosms inoculated by T1 consortium.

The comparison of the bacterial community diversity between non-inoculated and inoculated microcosms revealed that the inoculation with the T1 methane-oxidizing consortium significantly affected the richness, evenness, and the phylogenetic diversity of the bacterial communities. This was evident when comparing the overall diversity of the soil microbiota in control soil and during the incubation period following inoculation (Figure 5). The diversity levels observed in inoculated soils at zero week, one week and two week intervals were comparable, as indicated by equivalent richness and evenness metrics. These were using the observed species count, Simpson’s index, Shannon diversity indexes, and Chao1 estimator of species richness.

The diversity levels observed in inoculated soils at zero week, one week and two week intervals were comparable, as indicated by equivalent richness and evenness metrics. These were using the observed species count, Simpson’s index, and Shannon diversity indexes and Chao1 estimator of species richness.

Interestingly, at the four week sampling point, the inoculated soil samples clustered with other inoculated variants based on Chao1 and Observed indices, indicating comparable richness and evenness. However, they clustered with the control soil samples when assessed using Shannon and Simpson indices, suggesting that the time factor did not alter the overall diversity of species in the microbial community, but influenced the abundance and dominance of specific taxa. These findings underscore the nuanced effects of inoculation and incubation duration on soil microbial community structure and function.

**Figure 6:**
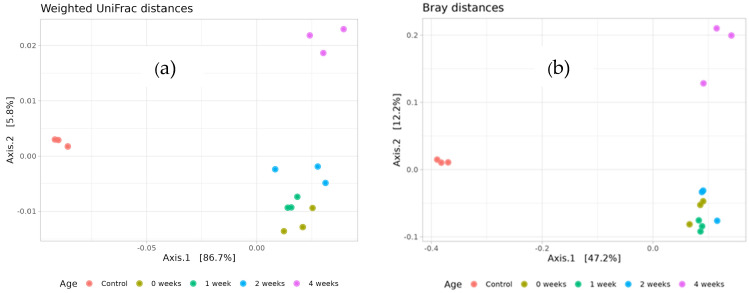
Similarity of soil microbial communities based on nonmetric multidimensional scaling of Weighted UniFrac distances (**a**) and Bray–Curtis values (**b**).

The microbial communities from the zero week, one week, and two week variants exhibited high similarity to each other, as indicated by both UniFrac and Bray–Curtis dissimilarity analyses (Figure 6). These communities clustered closely together, suggesting a consistent microbial structure during this early period. In contrast, the four week variant showed significant divergence from the zero week, one week, and two week groups, indicating substantial changes in microbial composition over time. Alterations in native microbial populations following inoculation may result from a variety of environmental and biological factors and warrant further investigation to fully understand the underlying mechanisms. By outcompeting dominant taxa and promoting competitive release, inoculation may boost biodiversity. On the other hand, if an inoculant’s establishment enhances the availability of resources, it can encourage the colonization of taxa that are already present in the species pool. Moreover, inoculation may facilitate the recruitment or emergence of previously unidentified bacterial taxa, potentially through shifts in resource availability, niche creation, or microbial signaling.

Considering survival of microbial inoculant in soil, we searched for OTUs displaying differential abundances between different treatments and duration of incubation experiment. We have used Log2 fold change as a way to express the different levels between two conditions (e.g., before and after treatment, or between different time variants) using a logarithmic scale. Log2 fold change (log2FC) in microbial communities refers to the logarithm (base 2) of the ratio of a microbial taxon’s abundance in one condition or sample compared to another.

**Figure 7:**
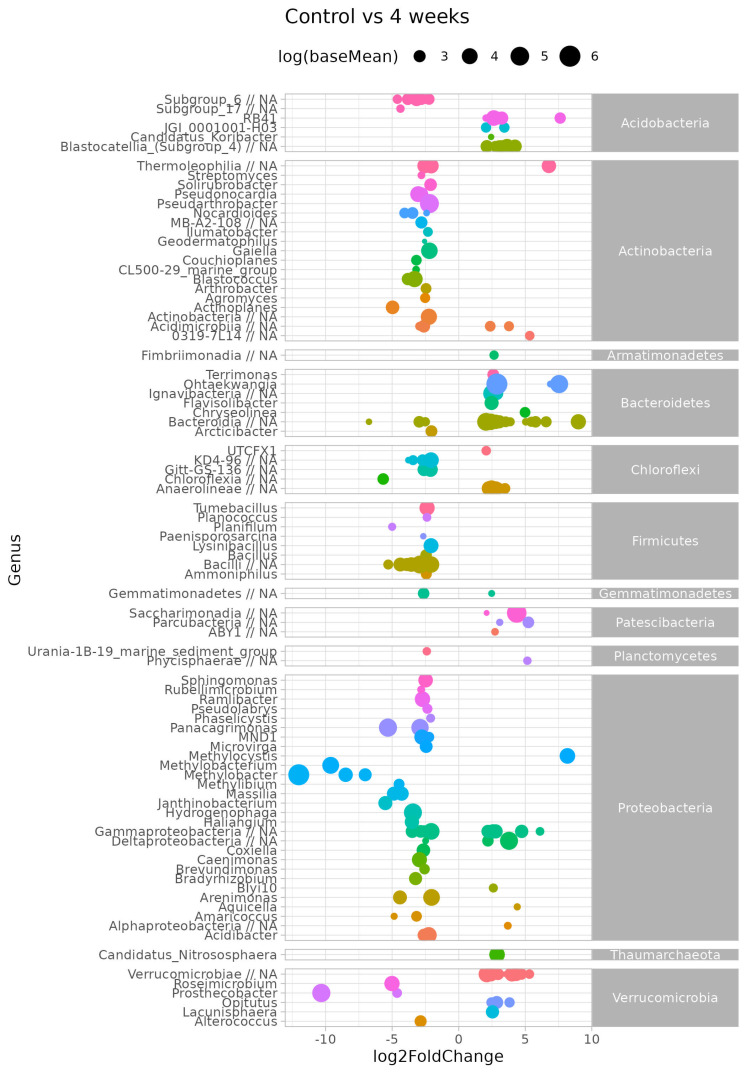
DESeq2 bar plot showing the log2-fold-change values (*x*-axis) of bacterial abundance at the genus level in control soil and four week variant.

This analysis was performed on filtered data, and low abundant taxa across all samples were excluded. Next, counts of ASV belonging to the same genus were summed to find general trends for the complete genus. Figure 7 shows the log-transformed log2 fold change (log2FC) of the differentially abundant genera between the resident soil microbial community (control) and after four weeks, after bacterial inoculant introduction (four week variant). A positive log2FC means a higher abundance for that genera in four week versus control, whereas a negative log2FC means a lower abundance for that genera in four week versus control.

Although we found no significant difference between the overall bacterial communities, several genera had different abundances. The genera with highest baseMean (≥3) were RB41 (Acidobacteria), *Thermoleophila* (Actinobacteria), *Ohtaekwangia* and unc.*Bacteroidia* (Bacteroidetes), *Anaerolinea* (Chloroflexi), *Saccharimonadia* and *Parculabacteria* (*Patescibacteria*), *Methylocystis* (Proteobacteria).

We compared the presence and relative abundance of bacterial phylotypes from the T1 consortium in the soil microcosms.

**Figure 8:**
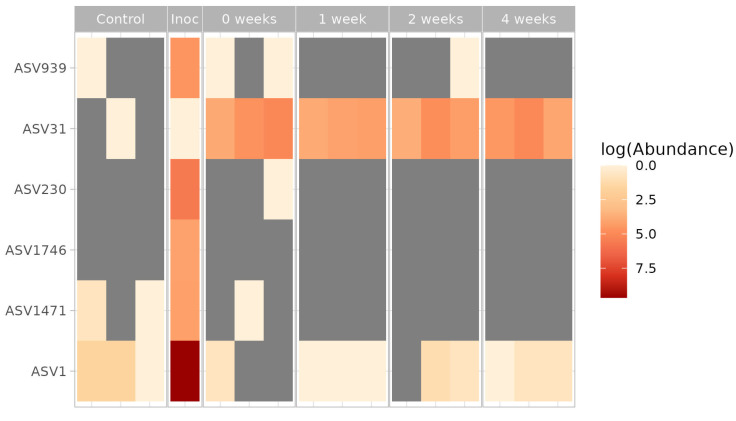
Heatmap of the relative abundance of bacteria displayed according to the proportion of the corresponding 16S rRNA gene fragments retrieved from each microcosm sample and inoculum. Abbreviations: control- without inoculation; inoc—inoculant T1 consortium; 0 week, 1 week, 2 weeks, 4 weeks—inoculated microcosms immediately after inoculation, after one, two and four weeks, respectively.

The obtained results are presented in Figure 8. It has been established that *Methylocystis* (ASV31), the dominant member of the consortium, constitutes a significant part of the microbial community in inoculated soils but is not found in the resident soil community. Two other *Methylocystis* ASV from inoculum were not found in the inoculated soil microcosms.

## 4. Discussion

Methanotrophic activity in soil is extremely important, not only from the point of view of the greenhouse potential of CH_4_, but also from the ecological point of view. Methylotrophs, including methanotrophs, are involved not only in methane cycling, but also in phosphorus acquisition, nitrogen fixation, phytohormone production, iron chelation, and plant growth promotion [34].

While the methane oxidation capacity of agricultural soils has recently received more focus, this environmentally significant phenomenon remains poorly understood. Moreover, there is a scarcity of data regarding the identification of methanotrophic bacteria in both agricultural and uncultivated soils, which is crucial for a complete understanding of CH_4_ oxidation.

Modern agricultural practices, aimed at achieving maximum yield, often lead to significant soil pollution. This makes the presence of methanotrophic bacteria in soil critically important, as their methane monooxygenase (MMO) enzymes can degrade various organic pollutants. Specifically, MMO is known to transform diverse hydrocarbons (alkanes, alkenes) and oxidize a broad spectrum of compounds, such as aliphatic hydrocarbons, alicyclic hydrocarbons, aromatic compounds (e.g., halogenated benzenes, toluene, styrene), and halogenated aliphatic compounds (including chloroform, dichloroethene, trichloroethylene, tetrachloroethene, and hydrochlorofluorocarbons) [35].

Various approaches exist for stimulating methane oxidation in soil, including the introduction of active methanotrophic consortia. Successful implementation of this approach requires obtaining an active bacterial inoculum and assessing the feasibility of its effective introduction into the soil. This study aimed to address these specific issues. As of right now, we are not aware of any studies on the introduction of methanotrophs into aerated soils, despite the fact that they are particularly important for controlling the amount of methane in the atmosphere.

Methanotrophic bacterial inoculation has so far produced favorable results for soils in habitats with high methane emissions. Particular focus is paid to rice fields, which are among the most important anthropogenic sources of methane. Methanotrophs filter methane generated in the deeper anoxic sediment layers in rice soils. Methane emissions were reduced by up to 44% in pot studies when methanotrophs-inoculated biogas-digesting effluent was applied [19]. In addition to lowering methane emissions, methanotrophs inoculation increased rice yield. For instance, in field experiments, co-inoculation of the plant growth-promoting bacteria *Paenibacillus polymyxa* MaAL70 with the methanotroph *Methylobacterium oryzae* MNL7 decreased methane emissions by up to 12.03% and raised rice yield by up to 14.04% [36]. According to another study, methane emissions from rice fields can be reduced by 60%, while rice yields can be increased by 35% [37].

The high-affinity methanotrophs in well-aerated soils, which act as atmospheric methane sinks, were the subject of our paper. Methanotrophic activity in the inoculated samples decreased during the incubation period, although it was still much higher than that found in the control soil: 17.4–13.7 ng CH_4_ g dry weight^−1^ h^−1^ and 0.9–5.2 ng CH_4_ g dry weight^−1^ h, respectively. Thus, inoculation with methanotrophs can also be applied to regulate the atmospheric methane sink.

The Chernevaya Taiga soil was chosen as the source for obtaining a methanotrophic inoculum. This decision was based on the understanding that forest soils are the main sink for atmospheric methane within terrestrial ecosystems [38]. Crucially, Chernevaya Taiga soils had not been previously investigated for their role in methane oxidation processes, making the data from our study particularly valuable. From samples of this soil, we successfully isolated a highly active and stable methanotrophic consortium for inoculating agricultural soil with naturally low methanotrophic activity.

Our laboratory experiments convincingly demonstrated the successful introduction of the T1 consortium. The methanotrophic activity of the inoculated samples remained significantly higher than the control soil for four weeks, and the number of introduced *Methylocystis* methanotrophs stabilized at a sufficiently high level. The long-term viability of introduced microorganisms in natural ecosystems is a critical and complex issue. Despite the extensive history of using bacterial fertilizers in agriculture, their effects are generally short-lived, and typically observed only during the growing season [39].

A key consideration when introducing foreign microorganisms into soil is their interaction with the indigenous microbiota and the resulting impact on microbial community composition. Introducing microbial inocula into non-sterile soil inevitably alters the existing microbial community structure. Since different soil types possess unique microbial communities, the effect of inoculation can vary significantly with the soil’s characteristics. Microbial diversity, which encompasses the range and abundance of microbial species, is a critical factor in soil ecosystem functioning.

The successful integration of methanotrophs into the soil microbial community hinges on fulfilling a set of conditions. First, the physicochemical soil conditions (pH, salinity, moisture, organic matter, porosity, etc.) must align with the needs of the exogenous methanotrophs, allowing them to locate and occupy suitable niches. Second, the inoculant must be competitive with indigenous microorganisms performing similar functions. Third, non-methanotrophic companion organisms that facilitate the introduction of methanotrophs can play an important role in the survival, growth, and activity of the introduced methanotrophs. Furthermore, active methanotrophic consortia hold potential for the biological remediation of soils contaminated by prevalent organic pollutants, including halogenated organic compounds.

## 5. Conclusions

The methanotrophic consortium T1, isolated from the soil of the Chernevaya Taiga, was successfully inoculated into agro-soil to restore its methanotrophic activity in laboratory microcosm experiments. Our findings indicate that the application of microbial inoculants not only specifically enhances methane oxidation activity but also significantly impacts the composition, diversity, and abundance of the soil microbiota.

Microcosm experiments provide valuable insights into the mechanism’s underlying microbial responses to environmental changes. Inoculating soil with methanotrophs presents a promising strategy for mitigating methane emissions from agricultural activities and contaminated sites. However, further research is essential to validate these findings in natural systems and to understand the broader ecological implications of introducing methanotrophs on methane cycling and soil health.

Improving the capacity of agricultural soils to sequester atmospheric methane remains a significant challenge in climate-soil research, due to complex biogeochemical interactions and the limited activity of methanotrophic communities in these environments. Future work will focus on using methanotroph biotechnology practically to address environmental issues. The research will continue in both the format of laboratory experiments with microcosms and in field experiments. In laboratory experiments, the long-term dynamics of methanotrophic activity and introduced methanotrophs, including the active part of the population, as well as changes in the native soil microbial community, will be tracked over a period of 12 months. In a field experiment, the methanotrophic activity and survival of the introduced methanotrophs will be evaluated over an annual cycle.

## Figures and Tables

**Table 1:** Top abundant bacterial OTUs and methylotrophic OTUs revealed by 16S rRNA genes analysis in Chernevaya taiga soil.

OTUs ID	Relative Abundance (%)	Taxonomy	Closest NCBI Match	Habitat
FS23_T-12	10.63	Cand. *Udaeobacter*	JQ367691.2	Pine plantation
FS23_T-3	7.61	*Unc.Gemmatimonadaceae*	PV095857.1	Forest soil
FS23_T-4	6.08	*Unc. Mycobacterium*	EF075152.1	Agricultural soil
FS23_T-6	5.04	*Xanthobacteraceae*_503485	JX505402.1	Rice field
FS23_T-7	3.36	*Neobacillus*	KX418947.1	Forest soil
FS23_T-8	2.76	Unc. *Chthoniobacterales*	EF664016.1	Agricultural soil
FS23_T-50	2.60	*Pseudolabrys*	FJ845160.1	Potato rhizosphere
Methanotrophs				
FS23_T-939	0.03	*Methylocysis*	J458502.1	Sewage sludge
FS23_T-16006	0.01	*Methylocella*	NR_027561.1	Forest soil
Methylotrophs				
FS23_T-5	3.10	*Methyloceanibacter*	FM956978.1	Rice field
FS23_T-91	0.11	*Hyphomicrobium methylovorum*	AB016812.1	Type strain
FS23_T-10911	0.01	Unc. *Methyloligellaceae*	KT790752.1	Karst area

## Data Availability

Raw sequences are uploaded to the SRA and available by PRJNA1285408 accession number, or directly via link: https://www.ncbi.nlm.nih.gov/sra/PRJNA1285408 (accessed on 2 July 2025).
